# An artificial neural network for estimating haplotype frequencies

**DOI:** 10.1186/1471-2156-6-S1-S129

**Published:** 2005-12-30

**Authors:** Kevin C Cartier, Daniel Baechle

**Affiliations:** 1 Case Western Reserve University, Department of Epidemiology and Biostatistics, Cleveland, OH, USA

## Abstract

The problem of estimating haplotype frequencies from population data has been considered by numerous investigators, resulting in a wide variety of possible algorithmic and statistical solutions. We propose a relatively unique approach that employs an artificial neural network (ANN) to predict the most likely haplotype frequencies from a sample of population genotype data. Through an innovative ANN design for mapping genotype patterns to diplotypes, we have produced a prototype that demonstrates the feasibility of this approach, with provisional results that correlate well with estimates produced by the expectation maximization algorithm for haplotype frequency estimation. Given the computational demands of estimating haplotype frequencies for 20 or more single-nucleotide polymorphisms, the ANN approach is promising because its design fits well with parallel computing architectures.

## Background

The relatively high cost of sequencing even short fragments of DNA means that, for the foreseeable future at least, the majority of research in genetic epidemiology will continue to be based on genotype data (i.e., marker phenotypes) produced by increasingly automated assay methods. This presents a fundamental problem to researchers in the field because current genotyping methods do not preserve phase information with respect to the individual whose genotype is being determined. In other words, the result of genotyping an individual may tell us that he has the two distinct alleles '*A*' and '*a*' at a given locus, but the respective maternal and paternal origin of each allele will generally not be known.

Many of the statistical methods of genetic epidemiology, and linkage analysis in particular, are specifically designed to measure the amount of association between a hypothesized genetic variant and the observed disease incidence within a sample of family or population data containing individual-specific genotype data. The availability of haplotype data would, of course, provide these methods with much greater power to detect linkage, particularly in the context of pedigree data. Because the haplotypes cannot be known with certainty it is reasonable to ask whether inferential methods might allow us to estimate haplotype frequencies either for a population or on an individual basis.

An elegant and intuitive "parsimony" algorithm [1] starts by identifying all genotypes that can be unambiguously resolved into an initial set of diplotypes (haplotype pairs), and maps them into a list of known haplotypes. Then for each of the remaining unresolved genotypes, the set of all diplotypes consistent with that genotype is constructed, and each haplotype pair is considered in turn. If one of the possible haplotypes can be found in the list of known haplotypes, then its corresponding (paired) haplotype is also added to the list. The process continues until no more genotypes can be resolved in this way. The main problems with the method are that it is guaranteed neither to start nor stop, and is susceptible to errors in the presence of multiple heterozygosity.

Another approach uses the well-known expectation-maximization (EM) algorithm to find the maximum likelihood of the haplotype frequencies over the given genotype data [2]. The method has been shown to be acceptably accurate and robust under a variety of scenarios [3], and noted deficiencies include its assumption of Hardy-Weinberg equilibrium proportions, relative slowness to converge, and difficulty in estimating errors.

In the Bayesian/Gibbs sampling method [4-6] the investigator first establishes a prior distribution (the Dirichlet or coalescent are two possibilities) of all haplotypes consistent with the set of given genotypes, and then produces empirical estimates of each individual's haplotype probability by repeatedly sampling from the posterior distribution created when that individual's genotype data is removed from the sample. This process is performed for all individuals, as many times as necessary, until some predetermined convergence criteria is met. As with all Bayesian methods, the main problem here is how to set the parameters for the prior distribution of haplotype frequencies.

In contrast to these methods, we considered an approach that treats haplotype frequency estimation as a type of multivariate classification problem, and in particular, our objective was to determine whether an artificial neural network (ANN) can be designed to recognize the most likely set of haplotypes – and hence their relative frequencies – that underlies the observed genotype data for a given population of individuals (we have deferred consideration of pedigrees to another time).

## Methods

A neural network is a particular type of machine learning algorithm designed to imitate the way learning is believed to occur in the human brain [7,8]. Small, autonomous data transformation units called nodes are grouped together in different layers, and weighted interconnections are established such that the outputs of nodes from a previous layer become inputs into nodes in a successive layer. A particular data signal, or pattern, is presented to the input layer and allowed to propagate through the network until it reaches the output layer, whereupon the network's response to the given pattern is revealed. During the network training phase, each observed response is compared with the expected output – the target – for a given pattern. The difference between the observed and expected response is computed to obtain the error for that presentation, which is then used to improve the network's performance by means of error back-propagation.

**Figure 1:**
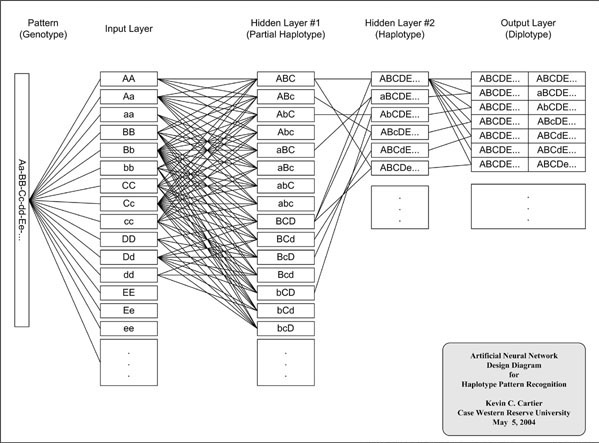
Artificial neural network design for haplotype pattern recognition.

Our goal was to construct an artificial neural network that could be trained to generate acceptably accurate haplotype classifications from a given set of genotype data. The first problem we needed to solve was how to design the different network layers such that a particular genotype pattern can be mapped to one or more diplotype classifications consistent with the pattern. We chose a design consisting of two hidden layers as shown in Figure 1. The network is designed to accept an arbitrary genotype pattern presented at the input layer, whose nodes are encoded as locus-specific genotypes. The network outputs represent diplotypes (i.e., haplotype pairs) consistent with the genotype data found in the population being studied, and the essential operation of the network is as follows:

1. During the feed-forward process, a given node of the input layer is activated only if its respective genotype occurs in the input pattern.

2. The first hidden layer, HL-1, consists of nodes representing three-locus partial haplotypes, and the weighted output of an activated input layer node is transmitted to a particular HL-1 node if either of the two alleles represented by the input layer occurs in the partial haplotype of the HL-1 node.

3. The second hidden layer, HL-2, consists of complete haplotypes (with respect to the set of loci implied by the input genotype patterns), and the weighted output of an activated HL-1 node is fed into a particular HL-2 node if the partial haplotype represented by the HL-1 node occurs in the complete haplotype of the HL-2 node.

4. Finally, the output layer consists of nodes representing possible diplotypes, and their nodes are activated by weighted output of HL-2 nodes in a fashion similar to that of the other layers.

Once the network has been created, and some data encoding scheme has been established for the input layer, the network must be trained so that the collection of weights and biases at each node result in optimal mappings between input patterns and output targets. Normally, a neural network should be trained by providing sets of data in which the correct classification is known for each input pattern, but since the true distribution of haplotypes for a given population is unknown, the network must be trained against the probability distribution of haplotypes that are consistent with the observed genotype data. For the purposes of this study, we arbitrarily assumed that each of the possible diplotypes consistent with a given genotype has an equal probability of being the correct classification.

The basic algorithm is as follows:

1. Obtain a pattern genotype by random sampling, without replacement, from the given population data.

2. For the given pattern, determine a target diplotype by random sampling from the uniform distribution of all diplotypes consistent with the selected genotype.

3. Present the pattern to the ANN input layer and propagate it forward to obtain one or more prospective (i.e., "predicted") diplotype classifications.

4. Compute the hamming distance (i.e., the number of locations at which two given diplotypes differ) between each of the prospective diplotypes and the target diplotype to obtain the pattern error.

5. Propagate the error backwards through the ANN to adjust the internodal weights.

6. If unsampled population data remains, go to step 1. Otherwise, the total error for the current training epoch is equal to .

7. If total error is less than some predetermined threshold, save current weights and stop. Otherwise, reshuffle the population data and begin another epoch at step 1.

After the ANN has been sufficiently trained, the network's training mode functions are disabled to allow the network to simply act as a pattern classifier as follows:

1. Make a final sequential pass through the original genotype data, in which each individual's genotype is presented to the ANN for classification.

2. Record the observed diplotype classification(s) for each presentation, and continue until each individual's genotype has been submitted for classification.

3. Produce the final estimates by converting the diplotype counts into relative haplotype frequencies.

## Results

We implemented our ANN design using PYTHON, a cross-platform programming language that is well suited to rapid development of software prototypes [9]. Our data sample consisted of 2,047 individual records randomly selected from REP01 of the combined Aipotu, Danacaa, and Karangar datasets (Genetic Analysis Workshop 14, Problem 2). We selected the first 10 loci for analysis, and recoded the alleles from 1/2, 1/2, 1/2, ... to A/a, B/b, C/c, ..., respectively.

**Figure 2:**
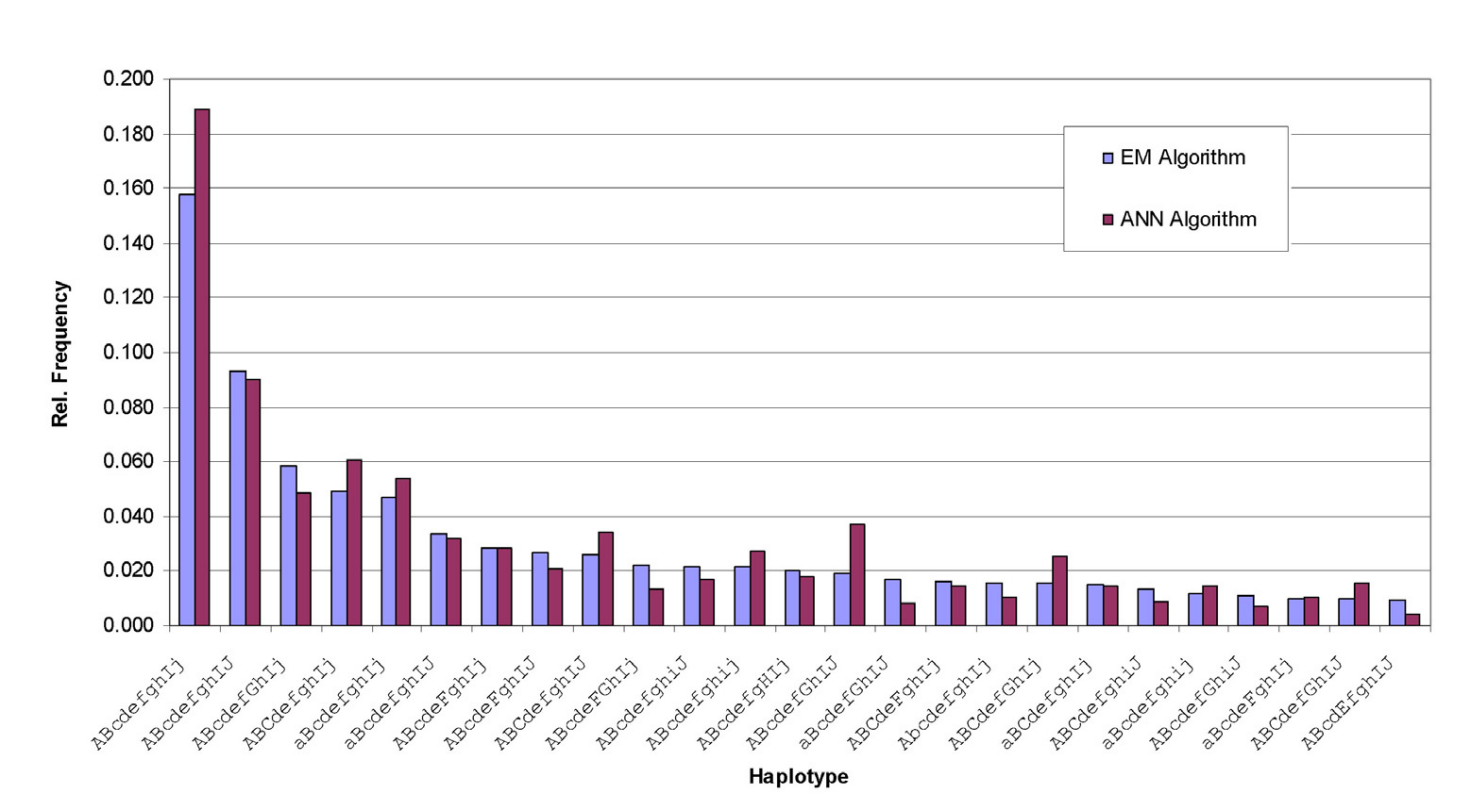
Comparison of algorithms for the 25 most frequently occurring haplotypes.

During the training phase, we allowed the ANN weights to train for more than 50 epochs, and obtained a minimum total error of 8.3. Classification was subsequently performed using the weights associated with the lowest total error. The simulated data did not include haplotype information for individuals and we were therefore unable to perform a proper error analysis on the ANN's performance. Instead, we simply compared the results of the ANN-based frequency estimates against estimates obtained from a C++ implementation of the EM algorithm for haplotype frequency estimation. The results were encouraging (see Figure 2). The correlation between the two independent methods was 0.98 for the most 100 frequent haplotypes, although the correlation decreases significantly when only low-frequency haplotypes are considered.

## Discussion

To the best of our knowledge, the ANN design shown in Figure 1 is a previously untried innovation for the haplotype estimation problem, and the provisional results we have obtained suggest that this approach may be worth further examination. Several shortcomings of the existing implementation should be considered then, any one of which could account for the observed correlational breakdown between the two estimation methods. First, the assumed uniform distribution of haplotype frequencies (given genotype) is almost certainly unrealistic, and efforts to improve the sampling scheme from that standpoint will likely prove beneficial. Secondly, our decision to halt training after 50 epochs was also somewhat arbitrary, and it is entirely possible that we could have obtained even better results had we allowed the minimum total error to drop closer to zero. Also, we arbitrarily designed the first hidden layer to contain nodes of three-locus haplotypes, but it would be interesting to see if a five-locus design produced significantly different results. Finally, the sample we analyzed did not reflect missing data at any of the selected single-nucleotide polymorphisms loci; however, it is our belief that the random sampling aspect of the method's algorithm would make it fairly robust in the presence of moderate amounts of missing genotype data.

We designed and tested the ANN to facilitate haplotype estimation from population data, deferring consideration of pedigrees to another time. A logical extension to the current ANN design, therefore, would be to allow for any familial relationships contained in the data sample. It is worth noting that the ANN's design comprises functionally independent nodes and layers, suggesting that it can be subdivided into smaller "subnetworks" that operate in parallel within some distributed processing framework. For example, given access to a networked cluster of 20 CPUs, the problem of reconstructing haplotypes for 20 SNPs could in principle be reduced from a single task with 2^20 ^possible solutions to a set of 20 parallel tasks, each with only two possible solutions to consider. Lastly, the lack of haplotype information for the simulated data precluded the usual type I error/power analyses, and these properties will of course need to be determined for this method.

## Conclusion

Our investigation has successfully demonstrated that an artificial neural network can be employed to estimate haplotype frequencies from population genotype data, and we have obtained provisional results that appear to correlate well with estimates produced by the EM algorithm for haplotype frequency estimation. On the other hand, the ANN presented here is at best a prototype, and there is clearly much room for improvement, both in its design and implementation. ANNs, by their nature, are well suited to parallel computing architectures and we therefore believe this approach to be worthy of further study.

## Abbreviations

ANN: Artificial neural network

EM: Expectation maximization

## Authors' contributions

KCC designed and implemented the artificial neural network described herein, performed all analyses, and wrote the manuscript. DB wrote and operated the C++ implementation of the EM algorithm for haplotype frequency estimation, and also prepared the simulated GAW data sample for analysis by each of the programs.
